# Blakesley’s Method of Filling Teeth

**Published:** 1858-04

**Authors:** 


### DR. BLAKESLEY'S METHOD OF FILLING TEETH.

Little over three years ago, Dr. A. Blakesley, of this city, caused some Foil to be made of our Crystal Gold. This Foil so pleased him that from that time till we commenced its manufacture, (one year ago), he continually urged us to make Gold Foil, averring that it was by far the best he had ever used. He occasionally had his Foil thus prepared until we undertook its manufacture, since which time he has used it *exclusively;* thus having a three years experience of its qualities.

To aid us in answering the many questions which are asked of us in reference to the method of using Crystal Gold Foil, we have requested Dr. Blakesley to condense for us the remarks made by him at the Dental Convention in Boston, last August.

Other modes of manipulation may be equally successful. This is given merely as *one entirely successful* method of using our Crystal Gold Foil, by a gentleman who stands deservedly high in the Dental Profession, and by whom the valuable properties of this article were first discovered; and not with the view of superseding any plan which any operator may have found to answer his purpose.

"It may be laid down as a maxim, in filling teeth, that the cavity, in the first place, should be perfectly freed from all decay, and that it should be of such a shape as will prevent the material used for filling from escaping. In order to secure this result, it is necessary that the interior of the cavity should be of larger dimensions than the orifice. The Crystal Gold Foil can be welded, in such a cavity, into so solid a mass that it is impossible to separate or displace it except by violent means. It will not crumble and fall away in particles, *if properly inserted*, which all other gold foils, (according to my experience), are liable to do. In order to secure this solidity, I deem the use of comparatively small instruments essential, as by their use the newly introduced portions of foil may be more thoroughly incorporated with that already in the cavity.

"In describing my mode of preparing the tooth, I shall, in order to adapt the description to the various positions of cavities, use the term bottom to indicate that surface of the cavity which is opposite the opening, whether that opening be upward, downward or lateral. After having thoroughly cleansed the cavity, I proceed to give it the shape as before stated; which I accomplish with small English broaches of suitable length, by slightly drilling or perforating the anterior and posterior angles of the upper part of the cavity, and also in the lower angle. I then connect them by drilling along the circumference between the enamel and the bony structure of the tooth, and also by means of a diamond pointed excavator, cutting in the same manner; thus forming an entire groove around the cavity. In cases where the walls of the cavity are frail, I rely almost entirely on these grooves for the retention of my filling, making them of considerable depth, so that when the filling is introduced, it is literally dove-tailed; the portions of gold occupying these grooves form projections from the main mass, with which they are thoroughly incorporated, thus rendering escape impossible.

"To prepare my gold for filling, I take a sheet of Foil, (say of No. 4), and with my hands perfectly dry, lightly roll it in into the shape of a rope, I take halves, thirds and quarters, and treat in the same manner. These I cut into square lengths, and thus have a series of pellets, from moderate size down to that of a small pin head.

"I commonly require about three sizes of filling instruments, turned at different angles, to suit any and every case that may be presented; the points of those in most constant use, will just enter respectively Nos. 22, 24 and 26 of the wire guage. The end of the smallest is cut with a screw-head file, into three well defined, rather delicate points, not too deep; and that of the larger into four points, observing the same precaution concerning the depth, or else the instrument will not disengage itself without tearing away part of the gold, while attempting to introduce it, thus choking the instrument and rendering it useless until freed of its burden.

"I commence to fill by inserting as large a pellet of gold as will occupy the upper part of the cavity; and continue adding pellets from side to side, until one-half of the bottom is covered; I then apply moderate pressure by direct and lateral force till the whole is tolerably condensed. I then endeavor to free it from the wall of the cavity at its edge. If I find I can do this, into the artificial cavity thus formed, I introduce other small pellets until it is completely filled—hard and solid. I repeat the operation on the opposite edge, and then firmly condense this portion of the filling, which now lies immovable and in a fit condition to be welded upon. I then proceed to fill the other half of the cavity, in the same manner, commencing from the lower part of the cavity, taking great precaution to have this portion well filled, as it is one of the most important points; and make firm the line of junction of the two halves, by forcing in small pellets wherever I can make a depression. I then weld on particles of gold until the cavity is filled up flush with the surface of the tooth, and when it is desirable, add on in such direction and quantity as to restore the original outline. In some cases of longitudinally fractured incisors, I have thus built on an addition of one-third or one-half of the original width of the tooth. My patients often express great delight at this perfect restoration of the shape of their teeth.

"The above is more particularly a description of cavities in a lateral position; in front incisors the same principle, however, holds good in all other cases, simply varying their position. When I have a very large cavity, for instance in a molar tooth, I sometimes commence by inserting whole, half or quarter sheets of foil, lightly formed into pellets of an oval or globular shape. In such cases, I frequently use a wedge shaped instrument, serrated at the point; finishing off as in ordinary cases with smaller pellets. I conclude the operation as usual, by polishing, "giving a surface," which this Foil receives to an eminent degree.

"Some operators have said that the Crystal Gold Foil hardens too quickly. I experience no trouble from this source, as I avoid applying sufficient pressure to render it unyielding until I am ready to condense it.

"It is important to avoid, when this Gold is used, a rounded shape of the cavity, as such a shape has a tendency to allow a pellet to escape from under the instrument, when you attempt to apply another one to it, while an angular shape serves to prevent this.

"To sum up, I would say, avoid the use of too large instruments, avoid introducing the Gold in too large masses, and avoid applying pressure till the Gold is where you want it to condense and harden.

"I consider the property of welding, which A. J. Watts & Co.'s Crystal Gold and Crystal Gold foil possess, as adding greatly to their value to the dental operator. It enables him to restore the lost shape of a tooth, and by taking care to secure proper points of support, to fill cavities and save teeth incapable of being reached by other Foils."

We will add that neither Dr. Blakesley, nor any other Dentist with whom we have conversed, finds it necessary to anneal our Foil just previous to use, as it is already in a fit state to weld when it is received from the manufacturers.

We can supply such instruments as Dr. BLAKESLEY uses, to those of the profession who desire them.

UTICA, November 1, 1857. A. J. WATTS & CO.

We publish the foregoing, believing it to be of great practical importance to the Profession. Dr. Blakesley is one of the oldest operators in the country, who in the use of Crystal Gold and annealed Foil has been eminently successful; his remarks descriptive of his mode of filling were listened to with profound attention, both before the American Convention in Boston, and the Mississippi Association in Cincinnati, because they were *practical* not theoretic.

Dr. Blakesley spent some two weeks with us in February last, during which time he went to our shop, stood by and directed and assisted *Mr. Sherwood,* until every variety of his points for pluggers were PERFECTED. We made a set of pluggers for Dr. B., himself, and are now prepared to supply the profession with Dr. B.'s patterns, in any size or style of handles desired, viz: in steel, ebony or ivory. The *set* consists of 28 instruments, viz:

4 Small wire handles, worth $1,50 per dozen.

3 Burrs for dressing fillings.

2 Pluggers with handles as may be desired.

The set as originally made, was 18 pluggers in ½ inch handles, 3 of ⅝ handles, 4 of wire handles, and 3 burrs, in large ebony handle, such as are generally used for elevators and screws. We are now making them, 2 doz., of one kind of handles, and 4 of wire handles.

